# Emergency department use by oldest-old patients from 2005 to 2010 in a Swiss university hospital

**DOI:** 10.1186/1472-6963-13-344

**Published:** 2013-09-08

**Authors:** Sarah Vilpert, Hélène Jaccard Ruedin, Lionel Trueb, Stéfanie Monod-Zorzi, Bertrand Yersin, Christophe Büla

**Affiliations:** 1 Swiss Health Observatory, Espace de l’Europe 10, CH-2010 Neuchâtel, Switzerland; 2 Service of Geriatric Medicine and Geriatric Readaptation, Department of Medicine, University of Lausanne Medical Center (CHUV), Centre Leenaards de la mémoire, 1011 Lausanne, Switzerland; 3 Service of Emergency Medicine, University of Lausanne Medical Center (CHUV), rue du Bugnon 46, CH-1011 Lausanne, Switzerland; 4 Service of Geriatric Medicine and Geriatric Readaptation, Department of Medicine, University of Lausanne Medical Center (CHUV), Mont-Paisible 16, CH-1011 Lausanne, Switzerland; 5 Service of Geriatric Medicine and Geriatric Readaptation, Department of Medicine, University of Lausanne Medical Center (CHUV), CUTR Sylvana, CH-1066 Epalinges, Switzerland

**Keywords:** Emergency department, Elderly, Population aging, Health care utilization

## Abstract

**Background:**

Aging of the population in all western countries will challenge Emergency Departments (ED) as old patients visit these health services more frequently and present with special needs. The aim of this study is to describe the trend in ED visits by patients aged 85 years and over between 2005 and 2010, and to compare their service use to that of patients aged 65–84 years during this period and to investigate the evolution of these comparisons over time.

**Methods:**

Data considered were all ED visits to the University of Lausanne Medical Center (CHUV), a tertiary Swiss teaching hospital, between 2005 and 2010 by patients aged 65 years and over (65+ years). ED visit characteristics were described according to age group and year. Incidence rates of ED visits and length of ED stay were calculated.

**Results:**

Between 2005 and 2010, ED visits by patients aged 65 years and over increased by 26% overall, and by 46% among those aged 85 years and over (85+ years). Estimated ED visit incidence rate for persons aged 85+ years old was twice as high as for persons aged 65–84 years. Compared to patients aged 65–84 years, those aged 85+ years were more likely to be hospitalized and have a longer ED stay. This latter difference increased over time between 2005 and 2010.

**Conclusions:**

Oldest-old patients are increasingly using ED services. These services need to adapt their care delivery processes to meet the needs of a rising number of these complex, multimorbid and vulnerable patients.

## Background

The increase in emergency department (ED) visits observed in western countries has been attributed to several factors, ranging from the reduced number of ED facilities to demographic expansion. Increase in ED utilization rates has also been proposed as an important factor since the increase in the volume of ED visits outpaces population expansion [1-3].

Among ED users, elderly patients show the highest incidence rates of ED use. As their incidence rate is currently increasing [4,5], the aging of the population will likely have both quantitative and qualitative consequences on ED activity. Indeed, elderly patients differ from younger patients with respect to their health service needs. They are associated with a higher degree of emergency, stay longer in the ED, are more likely to be admitted to hospital, and are at a higher risk of experiencing adverse outcomes after discharge [6-8].

Several studies have investigated the increased presence of elderly patients in ED [9,10]. These studies focused primarily on the increase in patients aged over 65 years, without describing the evolution over time of the age-specific burden on ED use among this heterogeneous population. Until recently, the segment of the oldest-old population remained modest (less than 2.3% of the Swiss population in 2009). However, this situation will change in the next 20 years as people aged 85 years and over become the fastest growing segment of the elderly population in most developed countries. For instance, a 72% rise is expected in this population group between 2010 and 2030 in Switzerland. As a consequence, this part of the population will also constitute the fastest growing group of ED users, leading to an aging of the ED patient mix.

Providing data on age-specific burden and health service use by the elderly is useful for raising the awareness of health care planners concerning the impact of the impending demographic changes on the potential utilization of health resources. In addition, providing these data is also useful since it makes ED staff aware of the evolving needs of their patients.

To further investigate these issues and gain more insight into current trends in ED utilization by the oldest-old segment of the population, we undertook the present study with three aims: 1) to determine the trend of ED use between 2005 to 2010 in a tertiary teaching hospital by very old patients aged 85 years and over; 2) to compare their service use to that of patients aged 65-84 years during this period; 3) to investigate the evolution of these comparisons over time.

## Methods

### Study design, setting and data sources

In Switzerland, health insurance coverage is universal and access to emergency departments is essentially unlimited.

The study was approved by the Ethics Committee of Canton de Vaud and constitutes a descriptive, retrospective analysis of all ED visits by patients aged 65 years and over (65+ y) that occurred between 2005 and 2010 at the University of Lausanne Medical Center (CHUV) in Lausanne, a Swiss urban area. The CHUV functions as a first-level community hospital for the 300,000 inhabitants of the Lausanne area, and as a second- and third-level referral hospital for Western Switzerland (about 1 to 1.5 million inhabitants).

Patients presenting at the CHUV ED are first triaged by a trained nurse. About a fifth, who suffer from conditions more amenable to primary care, are examined as outpatients and are not forwarded to the ED. These outpatients are not included in this study. The remaining patients (~35,000 patients/year) are admitted to the CHUV ED and were eligible for this study.

Data were collected from two administrative databases named AXYA and Gyroflux, which are not freely available. Database access was granted by the management of the CHUV. Overall 98% of ED visits registered in the two databases could be matched through patient and stay identification numbers.

Demographic data for the population living in the area of Lausanne between 2005 and 2010 were obtained from the statistical office of the Canton of Vaud.

### Measures

All ED visits by patients aged 65+ y that occurred between January 1, 2005, and December 31, 2010, were included in the study (N = 56,162 ED stays).

The incidence rates for ED visits were estimated for the residents of Lausanne, the local catchment area of the CHUV as a first-level hospital. ED visits by patients living in this region accounted for 67% to 70% of all ED visits by patients aged 65+ y during the study period.

Variables considered in the analyses were age, gender, marital status, place of residence before ED visit (home, nursing home, transfer from a psychiatric, an acute care or a rehabilitation hospital), date and time of ED arrival and discharge, reason for ED visit, triage level, disposition at discharge (home, nursing home, hospital admission, death), and return to ED within 30 days after ED discharge.

The reason for the ED visit and the triage level are assessed on arrival using a local triage scale. Until late 2009, the ED used a local 115-items, previously validated, triage scale (Lausanne triage and priority scale [11]). This triage scale comprises 5 triage levels (1 to 5), reflecting the acceptable time delay before the patient should be assessed. Starting in 2010, the Swiss Emergency Triage Scale (92-items and 4 triage levels) was used [12]. Because of this change, comparison of this variable over time was performed only between 2005 and 2009.

For the purpose of the analyses, patients were classified into two groups: a) care required immediately or within 15 minutes (triage levels 1 or 2) vs. b) care required within more than 15 minutes (triage levels 3 to 5). In this study, the reasons for ED visit recorded from the Lausanne triage and priority scale were aggregated into groups, and only the first three most important categories were depicted. They are analyzed in a separate paper together with the prevalence of the different triage categories among age groups.

Length of ED stay in hours (mean and survival analysis) was determined by calculating the difference between the date and time of arrival at, and discharge from, the ED.

Patients returning to ED within 30 days after their discharge were identified by their patient ID number. ED readmission rate was calculated per year and an ED readmission was considered as such only when the ED return followed an ED visit in the same year.

### Statistical analysis

ED visits were compared for the years 2005 vs. 2010, and for 65-84 years (65-84 y) vs. 85 years and over (85+ y) age groups, respectively.

Data used in this study are exhaustive and volumes for ED visits represent the actual number of ED stays. Data were analyzed using descriptive statistics. The chi-squared test was used to compare ED visit characteristics between the two age groups in a given year. Percentage differences by age groups between 2005 and 2010 were tested using the Z-test for difference in proportions. Incidence rates and their 95% confidence intervals (CIs) were calculated according to time and age groups. Relative risks (RR) of incidence rates between age groups and years were also calculated.

Differences in mean length of ED stay were compared using the Z-test comparison of means, and differences in median length of ED stay were compared using the Wilcoxon rank sum test. To account for the skewed distribution of the length of ED stay, means were calculated excluding the last percentile of the distribution.

Survival curves were plotted to show probability of ED discharge over the first 48 hours of ED stay. Proportions for stays shorter than, or equal to, 48 hours, and excluding in-ED death, amounted to 95% in both 2005 and 2010. In-ED death and stays over 48 hours were censoring events in survival analyses. The log-rank test was used to estimate differences between age groups and between years. The level of statistical significance was set at a P value of < 0.05. Data were analyzed using SAS statistical software v. 4.2.

## Results

Overall ED activity for patients aged over 18 years increased from 27,473 visits in 2005 to 33,445 visits in 2010, corresponding to an average of 75 and 92 daily visits, respectively (22% increase). Over the study period, patients aged 65+ y represented about 30% of all adult ED visits. Between 2005 and 2010, the mean number of daily ED visits by 65+ y patients rose from 23 to 29 patients, a 26% increase (20% increase among patients aged 18-64 years).

**Figure 1:**
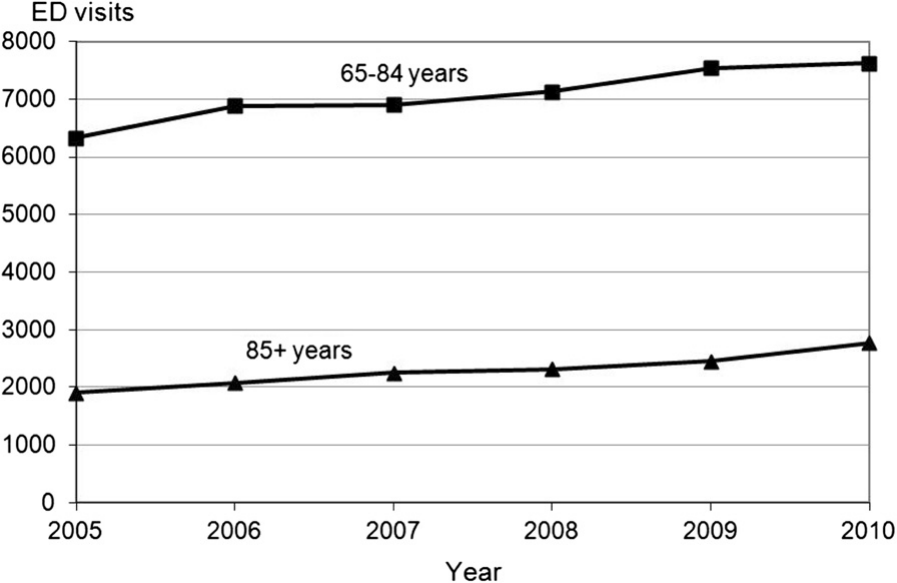
Evolution from 2005 to 2010 of emergency department visits by patients aged 65–84 years vs. 85 years and over.

Among ED 65+ y patients, the number of visits in those aged 85 years old and over (85+ y) increased from 1,901 to 2,772 visits, representing about a quarter of visits by patients 65+ y (2005: 23%; 2010: 27%). Thus, between 2005 and 2010, ED visits by 85+ y increased by 46%, a trend that accelerated between 2009 and 2010 (Figure 1). The median age of the 65+ y ED patients increased from 78.7 to 79.3 years. Anecdotally, ED visits by centenarians, mostly women, also increased from 12 to 21 patients between 2005 and 2010.

**Figure 2:**
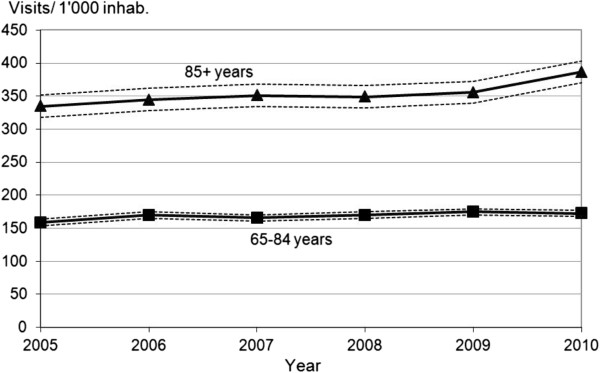
**Evolution from 2005 to 2010 of the incidence rate^a^ of Emergency Department visits by the permanent resident population of Lausanne^b^ by age groups.** Legend: ^a^ And its upper and lower 95% confidence intervals. ^b^ Includes districts of Lausanne and West Lausanne, Statistique Vaud 2012.

The incidence rate for ED visits increased slightly, but significantly, between 2005 and 2010 in both age groups (65-84 y: relative risk (RR) 1.09, 95% CI: 1.04-1.13, P = 0.040; 85+ y: RR 1.16, 95% CI 1.08-1.24, P = 0.033), producing incidence rates for ED visits of 173/1,000 inhabitants aged 65-84 y and 387/1,000 inhabitants aged 85+ y. In 2010, the RR of attending ED was 2.24 (95% CI 2.13-2.36, P < 0.001) times higher for the 85+ y than for the 65-84 y age group (Figure 2).

Comparisons of ED visit characteristics in 65-84 y and 85+ y patients, as well as their trends between 2005 and 2010, are provided in Table 1. During this period, despite the sizable increase in absolute numbers of ED visits in both age groups, there were little or no changes in the distribution of selected variables. Most of the 65+ y patients admitted to ED were still living at home (88% for 65-84 y, 82% for 85+ y in 2010). In 2010, 14% of the 85+ y patients who visited the ED were nursing home residents. The most common reason for ED visit involved cardiovascular problems in the 65-84 y patients and trauma in the 85+ y patients.

**Table 1:** Characteristics of emergency department visits (numbers and percentages) by patients aged 65–84 years vs. 85 years and over, 2005 and 2010

		**2005**			**2010**			**Z-Test 2005/2010 P value**	
		**65–84 years**	**85+ years**	**Chi2 P value**	**65-84 years**	**85+ years**	**Chi2 P value**	**65–84 years**	**85+ years**
**ED visits**^**a**^		6,327 (76.9%)	1,901 (23.1%)	*	7,618 (73.3%)	2,772 (26.7%)	*	*	*
**Sex**	**Male**	3,004 (47.5%)	602 (31.7%)	*	3,858 (50.6%)	963 (34.7%)	*	*	
	**Female**	3,323 (52.5%)	1,299 (68.3%)		3,760 (49.4%)	1,809 (65.3%)		*	
**Marital status**	**Married**	2,965 (46.9%)	432 (22.7%)	*	3,858 (50.6%)	796 (28.7%)	*	*	*
	**Other**^**b**^	3,362 (53.1%)	1,469 (77.3%)		3,760 (49.4%)	1,976 (71.3%)		*	*
**Residence**	**Home**	5,634 (89.2%)	1,582 (83.3%)	*	6,714 (88.2%)	2,262 (81.6%)	*		
	**Nursing home**	237 (3.8%)	228 (12.0%)		328 (4.3%)	370 (13.5%)			
	**Transfer**^**c**^	445 (7.1%)	90 (4.7%)		572 (7.5%)	139 (5.0%)			
**Triage level**^**d**^	**<= 15 min**	2,828 (44.7%)	841 (44.2%)		3,427 (45.4%)	1,078 (44.1%)			
	**> 15 min**	3,499 (55.3%)	1,060 (55.8%)		4,113 (54.6%)	1,369 (55.9%)			
**Reason for ED**	**Cardiovasc. conditions**	1,245 (19.7%)	344 (18.1%)	*	1,431 (19.0%)	389 (15.9%)	*		
**visit**^**d,e**^	**Trauma**	1,052 (16.6%)	497 (26.1%)		1,173 (15.6%)	583 (23.8%)			
	**Respiratory conditions**	829 (13.1%)	/		/	/		/	/
**Disposition**	**Hospital admission**^**c**^	3,871 (62.4%)	1,254 (68.4%)	*	4,478 (58.8%)	1,935 (69.8%)	*	*	
	**Home discharge**	2,127 (34.3%)	407 (22.2%)		2,893 (38.0%)	608 (21.9%)		*	
	**Nursing home discharge**	153 (2.6%)	145 (7.9%)		192 (2.5%)	198 (7.2%)			
	**In-ED death**	56 (0.9%)	29 (1.6%)		53 (0.7%)	30 (1.1%)			
**Length of ED stay**	**Mean ± SD**	12 h 58 m (±13.1)	14 h 46 m (±13.1)	*^f^	12 h 12 m (±12.8)	15 h 56 m (±14.8)	*^f^	*	*
**(hours and minutes)**	**Median**	6 h 52 m^h^	8 h 51 m^h^		6 h 37 m^h^	9 h 13 m^h^		*^g^	*^g^
**Return to ED within 30 days after ED**	**Discharge from ED to home/nursing home**	230 (3.6%)	51 (2.7%)		383 (5.0%)	95 (3.4%)	*		
**discharge**	**Discharge after hospital admission**	220 (3.5%)	63 (3.3%)		284 (3.7%)	118 (4.3%)			
	**No return**	5,877 (92.9%)	1,787 (94.0%)		6,951 (91.2%)	2,559 (92.3%)		*	*

Legend:

* P Value < 0.05.

^a^ED Emergency Department.

^b^Single, widowed, divorced, separated, unknown.

^c^Acute care, psychiatric and rehabilitation hospital.

^d^2009 instead of 2010.

^e^The three most common reasons for ED visit according to age group and year.

^f^Z-Test comparison of means.

^g^Wilcoxon rank sum test.

^h^Small differences with median length of ED stay reported in the survival analyses are due to censored stays (in-ED death and ED stays over 48 hours).

We also looked at disposition after ED stays. In 2005, 62% of ED visits by 65-84 y patients resulted in hospital admission (2010: 59%), mostly acute care, while 34% of patients returned home (2010: 38%). Despite this decrease in the rate of hospital admission between 2005 and 2010, the absolute number of hospitalizations for this age group increased by 607 patients. By comparison, among 85+ y patients, hospital admission was more frequent, approaching 70% in 2010, and home discharge from ED was less frequent (about 22% in both 2005 and 2010). From 2005 to 2010, an additional 681 ED visits resulted in a hospital admission in this age group. Overall, 3.5 additional patients aged 65 years or more were hospitalized each day in 2010 compared to 2005.

**Figure 3:**
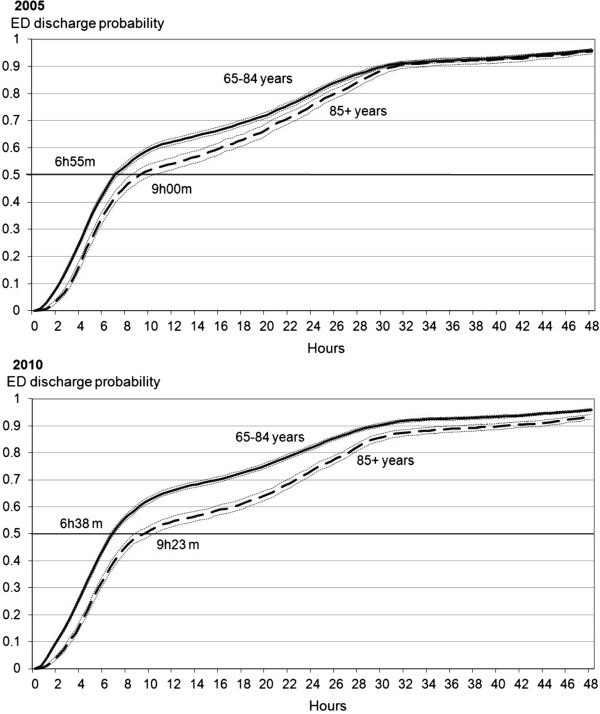
**Probability of Emergency Department discharge^a^ during the first 48 hours of patients aged 65–84 years vs. 85 years and over, 2005 and 2010.** Legend: ^a^ Home, nursing home or hospital admission. Log-rank comparing patients aged 65–84 years with patients 85 years and older in 2005: P value < 0.001. Log-rank comparing patients aged 65–84 years with patients 85 years and older in 2010: P value < 0.001. Log-rank comparing 2005 to 2010 among patients aged 65–84 years: P value = 0.003. Log-rank comparing 2005 to 2010 among patients aged 85 years and over: P value = 0.010.

Figures 3a and b show the probability of ED discharge (at home, nursing home or hospital admission) during the first 48 hours of stay. Patients aged 65-84 y had a shorter ED stay than their older counterparts in 2005 and 2010. Differences in median length of ED stay (50% of patients having already left the ED) between the two age groups averaged 2 hours 5 min in 2005, increasing to 2 hours 45 min in 2010. This increase resulted from inverse trends in length of ED stay in the two age groups, with a significant decrease among 65-84 y patients (from 6 hours 55 min to 6 hours 38 min, log-rank P = 0.003), and a significant increase among 85+ y patients (from 9 hours to 9 hours 23 min among 85+ y patients, log-rank P = 0.010). Sensitivity analysis without censuring death and stays over 48 hours provided similar results (results not shown).

## Discussion

This study provides original information on current ED use in the elderly population and highlights the growing importance of older patients in this health care setting.

A unique contribution of the current study is the provision of specific information about ED use among the oldest-old segment of the elderly population aged over 85 years. The volume of ED visits attributed to this population group is substantial, both overall and among the 65+ age groups where it accounts for a quarter of ED visits. Given the sharp increase in this specific segment of the elderly population expected in the next 20 years, the current aging of the elderly ED population is likely to show further expansion.

Besides the demographic expansion of the population aged 85+ y, this evolution also results from an increased incidence rate of ED use among this population over the timeframe covered by this study, a trend that was further confirmed in 2011 (data not shown). According to our estimated incidence rates for ED visits, inhabitants aged 85+ y were twice as likely to visit ED as inhabitants aged 65-84 y. This trend may reflect the chronological trend of disabled elderly persons to remain at home for longer rather than to be institutionalized. Alternatively, overuse for non-urgent conditions is sometimes mentioned to explain increased ED use in older persons [13,14]. However, the results from this study do not support this hypothesis, as there was no indication of any increased inappropriate use for non-urgent conditions or high readmission rates in the study population. Rather, there was a high proportion of patients in triage levels 1 and 2, i.e. requiring care within 15 minutes, a finding likely explained by the selective reorientation of some patients from triage levels 4 and 5 to the outpatient consultation service when felt appropriate.

Interestingly, incidence rates for ED use observed in this study were lower than those reported in previous studies (173 and 387/1,000 inhabitants aged 65-84 y and 85+ y, respectively, in this study vs. 450 and 820/1,000) [2,15,16]. The high density of primary care providers in Lausanne, with free access provided by the universal insurance coverage, as well as the good network of easily accessible home care services, may explain these lower figures observed in the current study. Nevertheless, these differences are unlikely to buffer the ongoing and future increase in the oldest-old ED population. Therefore, results from the current study further emphasize the need for ED staff to continuously adapt their process of care and acquire specific geriatric skills.

The current study expands previous findings that showed higher resource utilization and poorer outcomes in patients aged 65+ y compared to younger patients [4,6]. To our knowledge, this study is indeed the first to report the persistence of an age gradient in ED use among patients aged 65+ years. Moreover, this study extends our knowledge in highlighting diverging trends between youngest- and oldest-old patients over time in hospital admission and length of ED stay. Results show that, once presenting to the ED, oldest-olds patients were more likely to be admitted to the hospital than younger ones, a difference that increased over time from 2005 to 2010. Differences in hospital admission rates are essentially due to a decreased rate for patients aged 65-84 y, whereas this rate remained stable over time among those aged 85+ y. Likely, youngest patients may have benefited more from the shift from previously in-hospital activities and interventions to outpatient services. Further investigations are required to confirm this explanation. Of note, because of the overall aging of the population, despite the decreased admission rate observed in patients aged 65-84 y, the absolute number of admissions in this age group actually increased.

Similarly, this study provides original information on the chronologic trends in ED length of stay. Previous studies described longer ED stays for patients 65+ y when compared to younger patients [6,17-19]. The current study adds to these observations in showing that both age groups were significantly affected over time, but in opposite directions: while ED length of stay shortened in younger patients from 2005 to 2010, it lengthened in older ones. Hypotheses proposed to explain the differences observed between younger and overall 65+ ED users are likely to apply in the oldest-old age group. Firstly, medical evaluation of oldest-old patients is more complex and time-consuming [6,17]. Secondly, the lack of available hospital beds downstream may prolong ED stay [20]. In our study, the significant drop in length of ED stay observed in patients aged 65-84 y, combined with the decrease in their hospital admission rates after ED stay, may support this hypothesis. Thirdly, hospital beds are traditionally freed at the end of the afternoon, whereas about one half of older patients are admitted to ED between early morning and early afternoon [21]. Finally, discharges of oldest-old patients to their home or other health institutions require thorough and time-consuming coordination to ensure adequate transitions.

Nevertheless, attention should be paid to the evolving gap between both age groups with respect to length of ED stay over time. This trend may reflect an emerging mismatch between the services offered by ED units and the complex needs of geriatric patients [16,22,23]. Furthermore, ED structures may be deleterious for these patients when, for instance, limited access to natural light promotes delirium in cognitively impaired patients and a cluttered environment may represent a fall hazard [24].

ED ward managers should bear in mind that the ongoing increase in ED geriatric patient numbers will lead to an aging of their patient mix. Multimorbidity, functional, sensory and cognitive impairments will become prevailing issues, resulting in more heterogeneous care needs. These changes will have serious consequences on the organization and working procedures of ED teams, requiring urgent modification of the training curricula and care delivery process [16,25].

Only few studies have been conducted to optimize care provided in the ED to oldest-old patients. For instance, comprehensive geriatric assessment and management was shown in some studies to improve health outcome and decrease readmission in older ED patients discharged to their home [26-30]. Additional studies also investigated the benefits from best practices implementation within the ED to better meet the needs of the oldest-old patients with mixed results [31]. However, these studies focused mainly on health outcomes and incidence of specific geriatric conditions such as delirium or falls, rather than ED length of stay.

Our study presents several limitations. Firstly, this work was based on administrative databases that do not contain information on morbidity or investigations performed during the ED stay. Secondly, data are reported from a single institution and generalization is, as always, questionable. For instance, the higher proportion of high priority patients might result from local practice to re-orient some lowest priority patients to the outpatient clinic. However, trends observed in this study are much more likely to be related to demographic and epidemiological changes rather than the hospital’s practices and environment. Thirdly, as readmission rates were purposely calculated for each year separately, ED returns occurring in a different year than the year of first ED admission were missed, leading to a slight underestimation of the readmission rate.

## Conclusions

Our results highlight the growing importance of oldest-old patients in EDs. This trend results from the synergistic effects of an aging population and the increased incidence rates of ED use by the oldest-old population. Compared to their younger counterparts, e.g. patients aged 65-84 y, oldest-old patients show poorer outcomes in term of ED resource use, and the gap between both age groups seems to increase over time. The population aged 85+ y will be the fastest growing segment of the population over the coming years, with an absolute increase of about 50% by 2025. Even stable or slightly decreased ED visit incidence rates will not prevent the predicted acceleration of the aging case-mix in the ED population and the related changes in the care needs of ED patients. ED managers should be aware of these oncoming challenges so that they can adapt their training curricula and working procedures accordingly.

## Abbreviations

ED: Emergency department; CHUV: University of Lausanne Medical Center; RR: Relative risk; CI: Confidence interval; P: P Value.

## Competing interests

The authors declare that they have no competing interests.

## Authors’ contributions

SV, CB, HJR and SMZ conceived and designed the study. LT provided the data. SV acquired and managed the data and performed the statistical analyses. All authors interpreted the data. SV and HJR drafted the manuscript. CB and SMZ participated in critical revision of the manuscript’s intellectual content. All authors read and approved the final manuscript.

## Pre-publication history

The pre-publication history for this paper can be accessed here:

http://www.biomedcentral.com/1472-6963/13/344/prepub

## References

[B1] Pines JM Hilton JA Weber EJ Alkemade AJ Al Shabanah H Anderson PD Bernhard M Bertini A Gries A Ferrandiz S International perspectives on emergency department crowding Acad Emerg Med 2011 18 12 1358 1370 10.1111/j.1553-2712.2011.01235.x 22168200

[B2] Tang N Stein J Hsia RY Maselli JH Gonzales R Trends and Characteristics of US Emergency Department Visits, 1997–2007 Jama-J Am Med Assoc 2010 304 6 664 670 10.1001/jama.2010.1112 PMC3123697 20699458

[B3] Moskop JC Sklar DP Geiderman JM Schears RM Bookman KJ Emergency department crowding, part 1–concept, causes, and moral consequences Ann Emerg Med 2009 53 5 605 611 10.1016/j.annemergmed.2008.09.019 19027193

[B4] NHAMCS National Hospital Ambulatory Medical Care Survey: 2008 Emergency Department Summary 2008 Hyattsville: Ambulatory and Hospital Care Statistics Branch National Center for Health Statistics

[B5] NHAMCS National Ambulatory Medical Care Survey, 2005 Volume 21560 2005 Hyattsville: Ambulatory and Hospital Care Statistics Branch National Center for Health Statistics

[B6] Aminzadeh F Dalziel WB Older adults in the emergency department: a systematic review of patterns of use, adverse outcomes, and effectiveness of interventions Ann Emerg Med 2002 39 3 238 247 10.1067/mem.2002.121523 11867975

[B7] Gruneir A Bell CM Bronskill SE Schull M Anderson GM Rochon PA Frequency and pattern of emergency department visits by long-term care residents–a population-based study J Am Geriatr Soc 2010 58 3 510 517 10.1111/j.1532-5415.2010.02736.x 20398120

[B8] Roberts DC McKay MP Shaffer A Increasing rates of emergency department visits for elderly patients in the United States, 1993 to 2003 Ann Emerg Med 2008 51 6 769 774 10.1016/j.annemergmed.2007.09.011 18069088

[B9] Horney C Schmader K Sanders LL Heflin M Ragsdale L McConnell E Hocker M Hastings SN Health care utilization before and after an outpatient ED visit in older people Am J Emerg Med 2012 30 1 135 142 10.1016/j.ajem.2010.10.036 21216555 PMC3136637

[B10] Ionescu-Ittu R McCusker J Ciampi A Vadeboncoeur AM Roberge D Larouche D Verdon J Pineault R Continuity of primary care and emergency department utilization among elderly people CMAJ 2007 177 11 1362 1368 10.1503/cmaj.061615 18025427 PMC2072991

[B11] Hugli O Moujber M Simon J Geissbuhler A Yersin B Sarasin F Rutschmann O Analyse de la fiabilité et de la performance de deux échelles de tri à l’aide d’un simulateur informatique Journal Européen des Urgences 2008 21 A10723506959

[B12] Rutschmann OT Sieber RS Hugli OW Recommandations de la SSMUS pour le triage dans les services d’urgences hospitaliers en Suisse Bulletin des médecins suisses 2009 90 46 1789 179022849244

[B13] LaCalle E Rabin E Frequent users of emergency departments: the myths, the data, and the policy implications Ann Emerg Med 2010 56 1 42 48 10.1016/j.annemergmed.2010.01.032 20346540

[B14] McCusker J Cardin S Bellavance F Belzile E Return to the emergency department among elders: patterns and predictors Acad Emerg Med 2000 7 3 249 259 10.1111/j.1553-2712.2000.tb01070.x 10730832

[B15] Downing A Wilson R Older people’s use of Accident and Emergency services Age Ageing 2005 34 1 24 30 10.1093/ageing/afh214 15496462

[B16] Salvi F Morichi V Grilli A Giorgi R De Tommaso G Dessi-Fulgheri P The elderly in the emergency department: a critical review of problems and solutions Intern Emerg Med 2007 2 4 292 301 10.1007/s11739-007-0081-3 18043874

[B17] Gruneir A Silver MJ Rochon PA Emergency department use by older adults: a literature review on trends, appropriateness, and consequences of unmet health care needs Med Care Res Rev 2011 68 2 131 155 10.1177/1077558710379422 20829235

[B18] Yim VW Graham CA Rainer TH A comparison of emergency department utilization by elderly and younger adult patients presenting to three hospitals in Hong Kong Int J Emerg Med 2009 2 1 19 24 10.1007/s12245-009-0087-x 19390913 PMC2672983

[B19] Huang JA Weng RH Tsai WC Hu WH Yang DY Analysis of emergency department utilization by elderly patients under National Health Insurance Kaohsiung J Med Sci 2003 19 3 113 120 10.1016/S1607-551X(09)70458-9 12751871 PMC11917551

[B20] Moskop JC Sklar DP Geiderman JM Schears RM Bookman KJ Emergency department crowding, part 2–barriers to reform and strategies to overcome them Ann Emerg Med 2009 53 5 612 617 10.1016/j.annemergmed.2008.09.024 19027194

[B21] Carrasco V Baubeau D Les usagers des urgences: Premiers résultats d’une enquête nationale Etudes et Résultats. Direction de la recherche des études de l'évaluation et des statistiques (Drees) 2003 Paris

[B22] Sona A Maggiani G Astengo M Comba M Chiusano V Isaia G Merlo C Pricop L Quagliotti E Moiraghi C Determinants of recourse to hospital treatment in the elderly Eur J Public Health 2012 22 1 76 80 10.1093/eurpub/ckr008 21459840

[B23] Lakhan P Jones M Wilson A Courtney M Hirdes J Gray LC A prospective cohort study of geriatric syndromes among older medical patients admitted to acute care hospitals J Am Geriatr Soc 2011 59 11 2001 2008 10.1111/j.1532-5415.2011.03663.x 22092231

[B24] Hwang U Morrison RS The geriatric emergency department J Am Geriatr Soc 2007 55 11 1873 1876 10.1111/j.1532-5415.2007.01400.x 17916122

[B25] Prendergast HM Jurivich D Edison M Bunney EB Williams J Schlichting A Preparing the front line for the increase in the aging population: geriatric curriculum development for an emergency medicine residency program J Emerg Med 2010 38 3 386 392 10.1016/j.jemermed.2008.05.003 19028039

[B26] Graf CE Zekry D Giannelli S Michel J Chevalley T Efficiency and applicability of comprehensive geriatric assessment in the emergency department: a systematic reviewging Clin Exp Res 2011 23 4 244 254 10.1007/BF03337751 20930499

[B27] Ellis G Whitehead MA Robinson D O’Neill D Langhorne P Comprehensive geriatric assessment for older adults admitted to hospital: meta-analysis of randomised controlled trials BMJ 2011 343 d6553 10.1136/bmj.d6553 22034146 PMC3203013

[B28] Conroy SP Stevens T Parker SG Gladman JR A systematic review of comprehensive geriatric assessment to improve outcomes for frail older people being rapidly discharged from acute hospital: ‘interface geriatrics’ Age Ageing 2011 40 4 436 443 10.1093/ageing/afr060 21616954

[B29] Caplan GA Williams AJ Daly B Abraham K A randomized, controlled trial of comprehensive geriatric assessment and multidisciplinary intervention after discharge of elderly from the emergency department—The DEED II study J Am Geriatr Soc 2004 52 9 1417 1423 10.1111/j.1532-5415.2004.52401.x 15341540

[B30] McCusker J Verdon J Do geriatric interventions reduce emergency department visits? A systematic review J Gerontol A Biol Sci Med Sci 2006 61 53 62 10.1093/gerona/61.1.53 16456194

[B31] Schnitker L Martin-Khan M Beattie E Gray L What is the evidence to guide best practice for the management of older people with cognitive impairment presenting to emergency departments? A systematic review Adv Emerg Nurs J 2013 35 2 154 69 23636047 10.1097/TME.0b013e31828c7f4a

